# Epistasis analysis links immune cascades and cerebral amyloidosis

**DOI:** 10.1186/s12974-015-0436-z

**Published:** 2015-12-01

**Authors:** Andréa L. Benedet, Aurélie Labbe, Philippe Lemay, Eduardo R. Zimmer, Tharick A. Pascoal, Antoine Leuzy, Sulantha Mathotaarachchi, Sara Mohades, Monica Shin, Alexandre Dionne-Laporte, Thomas Beaudry, Cynthia Picard, Serge Gauthier, Judes Poirier, Guy Rouleau, Pedro Rosa-Neto, for the Alzheimer’s Disease Neuroimaging Initiative

**Affiliations:** Translational Neuroimaging Laboratory, McGill University Research Centre for Studies in Aging, 6825 LaSalle Blvd, H4H 1R3 Montreal, QC Canada; CAPES Foundation, Ministry of Education of Brazil, Brasília, Brazil; Douglas Hospital Research Centre, McGill University, Montreal, Canada; Department of Epidemiology, Biostatistics & Occupational Health, McGill University, Montreal, Canada; Department of Psychiatry, McGill University, Montreal, Canada; Department of Biochemistry, Université de Montréal, Montréal, Canada; Department of Biochemistry, Federal University of Rio Grande do Sul, Porto Alegre, Brazil; Brain Institute of Rio Grande do Sul (BraIns), Pontifical Catholic University of Rio Grande do Sul (PUCRS), Porto Alegre, Brazil; Department of NVS, Center for Alzheimer Research, Translational Alzheimer Neurobiology, Karolinska Institutet, Stockholm, Sweden; Alzheimer’s Disease Research Unit, McGill University Research Centre for Studies in Aging, McGill University, Montreal, Canada; Department of Neurology and Neurosurgery, McGill University, Montreal, Canada; Montreal Neurological Institute, Montreal, Canada

## Abstract

**Background:**

Several lines of evidence suggest the involvement of neuroinflammatory changes in Alzheimer’s disease (AD) pathophysiology such as amyloidosis and neurodegeneration. In fact, genome-wide association studies (GWAS) have shown a link between genes involved in neuroinflammation and AD. In order to further investigate whether interactions between candidate genetic variances coding for neuroinflammatory molecules are associated with brain amyloid β (Aβ) fibrillary accumulation, we conducted an epistasis analysis on a pool of genes associated with molecular mediators of inflammation.

**Methods:**

[^18^F]Florbetapir positron emission tomography (PET) imaging was employed to assess brain Aβ levels in 417 participants from ADNI-GO/2 and posteriorly 174 from ADNI-1. IL-1β, IL4, IL6, IL6r, IL10, IL12, IL18, C5, and C9 genes were chosen based on previous studies conducted in AD patients. Using the [^18^F]florbetapir standardized uptake value ratio (SUVR) as a quantitative measure of fibrillary Aβ, epistasis analyses were performed between two sets of markers of immune-related genes using gender, diagnosis, and apolipoprotein E (APOE) as covariates. Voxel-based analyses were also conducted. The results were corrected for multiple comparison tests. Cerebrospinal fluid (CSF) Aβ_1-42_/phosphorylated tau (p-tau) ratio concentrations were used to confirm such associations.

**Results:**

Epistasis analysis unveiled two significant single nucleotide polymorphism (SNP)-SNP interactions (false discovery rate (FDR) threshold 0.1), both interactions between *C9* gene (rs261752) and *IL6r* gene (rs4240872, rs7514452). In a combined sample, the interactions were confirmed (*p* ≤ 10–5) and associated with amyloid accumulation within cognitively normal and AD spectrum groups. Voxel-based analysis corroborated initial findings. CSF biomarker (Aβ_1-42_/p-tau) confirmed the genetic interaction. Additionally, rs4240872 and rs7514452 SNPs were shown to be associated with CSF and plasma concentrations of IL6r protein.

**Conclusions:**

Certain allele combinations involving *IL6r* and *C9* genes are associated with Aβ burden in the brain. Hypothesis-driven search for epistasis is a valuable strategy for investigating imaging endophenotypes in complex neurodegenerative diseases.

**Electronic supplementary material:**

The online version of this article (doi:10.1186/s12974-015-0436-z) contains supplementary material, which is available to authorized users.

## Background

Alzheimer’s disease (AD) is the most common form of dementia worldwide and has been recently reconceptualized as a dynamic and progressive process in which pathological changes start decades prior to the onset of clinical symptoms [1, 2]. According to the amyloid cascade hypothesis [3], the accumulation of brain amyloid β (Aβ) sets a cascade of progressive neurodegenerative changes—including the formation of intracellular inclusion of neurofibrillary tangles (NFTs)—resulting in cognitive impairment and, ultimately, dementia. Imaging and cerebrospinal fluid (CSF) biomarkers have successfully advanced our knowledge in terms of the evolution of AD [1]. However, the most recent hypothetical model of AD biomarkers [4] has not explored the role of neuroinflammation, a phenomenon implicated in the pathogenesis of AD by several lines of evidence [5–7].

It is becoming a common theme the high likelihood that neuroinflammation in AD is dependent on several genetic factors and is affected by environmental interactions that happen during an individual’s lifetime (for review, see [8]). Previous studies have shown important interactions between immune responses and brain amyloidosis [9], with both in vitro and in vivo studies demonstrating altered cytokine expression in AD. In addition, neuroinflammation secondary to systemic infections, traumatic brain injuries, or other neurologic conditions has been shown to increase the risk of sporadic AD [10, 11].

Currently, it is widely accepted that Aβ is associated with innate immunity pathways—as well as molecular mediators such as cytokines, chemokines, and complement molecules—leading to neuroinflammation and disturbance in brain homeostasis. However, findings linking immune-related genes with AD have raised the possibility that inflammation is the cause of brain amyloid load. In fact, the activation of the immune response by damage-associated factors is able to increase Aβ production (for review, see [12]). Thus, it has been hypothesized that impaired immune response either fails to clear Aβ from the brain or drives an overreaction against this protein, resulting in chronic inflammation, which effects could be either harmful or protective in nature.

Endophenotypes associated with variations in immune-related genes, particularly related to AD neuropathological features, remain elusive. Genome-wide association studies (GWAS) and meta-analysis have found immunogenetic variants associated with AD, namely *CR1*, *CLU*, *TREM2*, *PICALM*, *CD33*, and *MEF2C*, reasserting the role of the immune system in AD pathophysiology [13–17]. However, recent investigations did not reveal a link between brain amyloidosis and immunologic genetic variants [18, 19], suggesting that some endophenotypes might be affected by gene-to-gene interactions or epistasis.

In multifactorial diseases such as AD, the power to detect isolated genetic variants can be reduced due to epistatic effects, which occur when one locus masks or alters the effect of another [20–22]. In this respect, approaches moving beyond single-marker outcomes may better capture heritability links [23].

In this study, we aimed to investigate the interactions between immune-related genes—primarily molecular mediators of inflammation—and the accumulation of Aβ in vivo, as quantitated by amyloid imaging with positron emission tomography (PET). We hypothesize that differential amyloid burden is associated with the deregulation of innate immunity response, which could be evidenced by epistasis analysis of genes that encode for immune proteins reported to be related to AD.

## Methods

### Research subjects

Data used in the preparation of this article were obtained from the Alzheimer’s Disease Neuroimaging Initiative (ADNI) database (adni.loni.usc.edu). The ADNI was launched in 2003 as a public-private partnership, led by Principal Investigator Michael W. Weiner, MD. The primary goal of ADNI has been to test whether serial magnetic resonance imaging (MRI), PET, other biological markers, and clinical and neuropsychological assessment can be combined to measure the progression of mild cognitive impairment and early Alzheimer’s disease. To date, over 1500 adults with ages ranging from 55 to 90 years old participate in the research, consisting of cognitively normal (CN) older individuals, subjects with amnestic mild cognitive impairment (MCI), and individuals that met the NINCDS/ADRDA criteria for probable AD. Further details about inclusion and exclusion criteria can be found at the ADNI website (http://www.adni-info.org/Scientists/ADNIStudyProcedures.aspx).

All subjects included in the ADNI project provided written informed consent, according to Helsinki Declaration, at the time of enrolment for imaging and genetic sample collection and completed clinical symptom assessments approved by each participating sites’ Institutional Review Board. Following ADNI’s policies, the principal investigator of the present study has accepted ADNI Data Use Agreement and is authorized to use ADNI data.

This report is based on data acquired from 417 participants from ADNI-GO/2 and 174 from ADNI-1, from whom both genetic and PET data were available. Demographic data is summarized in Table 1.

**Table 1:** Demographic and key characteristics of the sample at baseline

	ADNI-GO/2			ADNI-1			Combined dataset		
	Cognitively normal (CN)	Mild cognitive impairment (MCI)	Alzheimer’s disease (AD)	Cognitively normal (CN)	Mild cognitive impairment (MCI)	Alzheimer’s disease (AD)	Cognitively normal (CN)	Mild cognitive impairment (MCI)	Alzheimer’s disease (AD)
Number of subjects (%)	123 (29.6)	266 (64.9)	27 (6.5)	73 (42)	58 (33.3)	43 (24.7)	196 (33.2)	324 (54.9)	70 (11.9)
Number of males (%)	62 (50.4)	146 (54.9)	17 (63.0)	36 (49.3)	38 (65.5)	25 (58.1)	98 (50.0)	184 (56.8)	42 (60.0)
Number of *ApoE ε4* carriers (%)	32 (26.0)^a^	118 (44.4)^a^	17 (63.0)^a^	19 (26)	21 (36.2)	27 (62.8)^b^	51 (26.0)^a^	139 (42.9)^a^	44 (62.9)^a^
Mean age (SD)	74.37 (5.62)	71.45 (7.61)^b^	75.56 (10.67)	80.73 (4.72)	79.64 (7.42)	76.47 (6.24)^b^	76.74 (6.12)	72.92 (8.19)^b^	76.12 (8.17)
Mean years of education (SD)	16.50 (2.64)	16.00 (2.55)	16.41 (2.32)	15.85 (2.92)	15.38 (3.17)	16.21 (2.66)	16.26 (2.76)	15.89 (2.67)	16.23 (2.49)
Mean CDR-SOB (SD)	0.03 (0.14)^a^	1.35 (0.86)^a^	4.70 (1.20)^a^	0.26 (0.8)^a^	1.73 (1.43)^a^	5.63 (3.28)^a^	0.12 (0.51)^a^	1.42 (0.99)^a^	5.26 (2.69)^a^
Mean MMSE (SD)	29.05 (1.16)^a^	28.23 (1.62)^a^	22.52 (1.88)^a^	29.03 (1.3)^a^	27.69 (1.88)^a^	21.44 (5.0)^a^	29.04 (1.21)^a^	28.13 (1.68)^a^	21.87 (4.13)^a^
Mean [^18^F]florbetapir SUVR (SD)	1.24 (0.20)^a^	1.31 (0.23)^a^	1.51 (0.24)^a^	1.15 (0.13)^a^	1.25 (0.18)^a^	1.40 (0.18)^a^	1.20 (0.19)^a^	1.30 (0.23)^a^	1.44 (0.21)^a^

The baseline considered here is the date of the first [^18^F]florbetapir PET acquisition

*SD* standard deviation, *CDR-SOB* Clinical Dementia Rating Scale Sum of Boxes, *MMSE* Mini-Mental State Examination, *SUVR* standard uptake value ratio

^a^Statistically different between all groups from the same sample

^b^Statistically different from the other groups from the same sample

### PET methods

Amyloid load was estimated using the [^18^F]florbetapir PET standardized uptake value ratio (SUVR). A detailed description of the [^18^F]florbetapir imaging acquisition protocol can be found online at the ADNI website. PET image processing and estimation of global SUVRs have been described previously [24]. All image processing, including generation of regions of interest, is summarized in Additional file 1: Figure S1.

### Gene selection

We have chosen to verify possible interactions between the main interleukins (IL) reported to be associated with AD pathology and proteins of the membrane attack complex (MAC). Selected MAC key proteins include complement 5 (C5) and complement 9 (C9). They are respectively the first and last proteins to be activated in the MAC cascade, and both are also found to be associated with amyloid plaques in AD brain [25, 26]. The interleukins selected were the most frequently reported to be related to AD [26–30]. IL1β, IL6 (and its receptor IL6r), IL12, and IL18 have shown to be differentially expressed in AD brain when compared to controls. These pro-inflammatory cytokines display increased expression in AD brains and/or are associated with amyloid plaques [28, 31–33]. They also seem to reduce AD-like phenotypes when inhibited in animal models [34, 35]. IL4 and IL10 have anti-inflammatory properties, and they all have been found to be associated with AD, either by in vitro studies, genetic studies, or biochemical analysis of plasma, CSF, and/or AD brains [27, 36].

### Genetic analysis

The ADNI-GO/2 subjects were genotyped using the Illumina HumanOmniExpress BeadChip (Illumina, Inc., San Diego, CA) array [37], while for ADNI-1 subjects, correspondent genotypes were obtained from HumanOmni2.5 BeadChip (Illumina, Inc., San Diego, CA). Quality control was performed using PLINK software (version 1.07) [38] excluding single nucleotide polymorphisms (SNPs) with a genotyping efficiency <95 %, a minor allele frequency of <5 %, or deviation from Hardy-Weinberg equilibrium <1 × 10^−6^. Subjects were excluded if they had a call rate <95 % and if genetic relatedness was detected (PI_HAT > 0.5). Population stratification was accounted for by subtracting the effect of the first two principal components using the Eigenstrat routine [39] in the Eigensoft V5.0 package [40] and posterior visualization of the Q-Q plots.

For the epistasis analysis, we selected 10 genes related to molecular mediators of inflammation as mentioned above. These genes were grouped into two sets according to their protein function, set 1 being composed of interleukins and one interleukin receptor (*IL-1β*, *IL4*, *IL6*, *IL6r*, *IL10*, *IL12*, *IL18*) and set 2 of proteins of the MAC (*C5*, *C9*). Using the UCSC Genome Browser (http://genome.ucsc.edu), we annotated the start and end points of each selected gene and all SNPs present within this region were obtained with PLINK. SNAP Proxy Search (version 2.2) [41] was used to verify and then remove markers in high linkage disequilibrium (*r*^2^ > 0.8). The remaining SNPs were grouped together within the respective set (31 SNPs in set 1 and 21 in set 2) as summarized in Additional file 2: Table S1. Epistasis analysis was performed using R [42].

### Cerebrospinal fluid concentrations of phosphorylated tau and amyloid-β_1-42_

Baseline CSF amyloid-β_1-42_ (Aβ_1-42_) and phosphorylated tau (p-tau) levels were measured using the multiplex xMAP Luminex platform (Luminex Corp, Austin, TX) and Innogenetics/Fujirebio AlzBio3 immunoassay kits. The methodology applied for aliquot collection, peptide quantification, as well as quality control and data normalization are described in previous reports [43, 44]. The normalized data was used to calculate the Aβ_1-42_/p-tau ratio, which is the phenotype used to confirm findings obtained with [^18^F]florbetapir.

### Cerebrospinal fluid and plasmatic protein levels

The Biomarkers Consortium CSF and Plasma Proteomics Project multiplex data are available for ADNI-1 subjects from whom protein levels were measured at the baseline for both biospecimens and 12-month follow-up visit only in the plasma. The description of the methodology regarding the sample acquisition, sample processing and analysis, as well as quality control procedures is available at the ADNI website (http://adni.loni.usc.edu/methods/biomarker-analysis/proteomic-analysis and http://adni.loni.usc.edu/data-samples/biospecimen-data/).

### Statistical analysis

The epistasis analysis was performed with R, in which a linear model was used to test the interaction between two SNPs in a given pair. Each SNP pair was composed of one SNP from each set, resulting in 651 pairs tested. Subjects’ genotypes were acquired using PLINK and categorized based on minor allele counts (additive model). The quantitative trait analyzed was the [^18^F]florbetapir SUVR. In the model, diagnostic status (AD, MCI, or CN), gender, and number of apolipoprotein E allele 4 (*ApoE ε4*) were added as covariates. False discovery rate (FDR) was used to correct for multiple comparisons. The significant interactions (after FDR correction at 0.1 level) found with the ADNI-GO/2 sample were tested for replication using both the ADNI-1 sample and the combined dataset (ADNI-1 and ADNI-GO/2). The comparison of the [^18^F]florbetapir SUVR means between genotype groups was performed using the combined dataset. Tukey’s honest significant difference (HSD) test was used in the post hoc analysis. The significant interactions were also tested within the two groups obtained from the combined dataset: CN and AD spectrums (MCI and Alzheimer dementia patients). In these late comparisons, the model applied followed the same criteria described for the analysis with the whole sample.

Voxel-based analysis was carried out to confirm volume of interest analysis. Parametric images were obtained using the methodology summarized in Additional file 1: Figure S1. First, the model was tested for the most significant interaction. Then, we compared the groups participating in the two most significant contrasts found in Tukey’s HSD test applied in the global SUVR analysis. Voxel-based statistical differences were obtained by contrasting the [^18^F]florbetapir SUVR between genotype groups, adjusting for gender, diagnostic status, and *ApoE ε4* using the RMINC imaging tool. RMINC is an imaging package that allows images files in the Medical Imaging NetCDF (MINC) to be analyzed with the powerful statistical environment R. After random field theory (RFT) [45] correction for multiple comparisons, the *T* value threshold of significance is ≥3.0 (*p* ≤ 0.05) for the interaction model and ≥3.2 (*p* ≤ 0.05) for the group comparison.

To confirm the association found with [^18^F]florbetapir phenotype, the most significant interacting pair of SNPs was tested using the baseline Aβ_1-42_/p-tau ratio as the dependent variable, which was available for a subsample of 208 subjects. The applied model had diagnosis, gender, and number of *ApoE ε4* as covariates.

The effect of the polymorphisms on their respective protein levels in the plasma was tested in ADNI-1 subjects with proteomic data available (*n* = 114). A linear mixed-effects model—adjusted for diagnosis and gender—was applied to analyze the effect of the genotype in both baseline and 12-month follow-up data. The genotypes were categorized according to the presence or absence of the minor allele. An additional linear model tested for associations between the polymorphisms and the CSF concentrations of their respective proteins (*n* = 81).

## Results

### Epistasis analysis indicates that the interaction between *C9* and *IL6R* genes is associated with brain amyloid deposition

After applying the quality control steps as previously described, one pair of subjects was found to be genetically related. One of the subjects was thus randomly selected and excluded from the study. No difference was seen in the Q-Q plots for SNPs as main effects, before and after adjustment for the first two principal components (data not shown). This suggests that our sample is genetically homogeneous. Epistasis analysis unveiled two significant SNP-SNP interactions after FDR correction (FDR threshold *p* = 0.1; see Table 2 and Additional file 2: Table S2).

**Table 2:** SNP-SNP interaction information

			ADNI-GO/2			ADNI-1		Combined dataset	
Gene interactions	SNP interactions	Minor allele	MAF	*p* value	*P* value adjusted^a^	MAF	*p* value	MAF	*p* value
C9* *IL6r*	rs261752* rs7514452	C C	0.44 0.18	1.0 × 10^−4^	0.06	0.43 0.19	0.06	0.44 0.20	1.1 × 10^−5^
C9* *IL6r*	rs261752* rs4240872	C C	0.44 0.24	2.1 × 10^−4^	0.07	0.43 0.25	0.06	0.44 0.24	3.7 × 10^−5^

*MAF* minor allele frequency

^a^FDR-corrected *p* value (threshold 0.1)

The most significant interaction was between the SNP rs261752 of the *complement 9* (*C9*) gene and the SNP rs7514452 annotated to the *interleukin 6 receptor* (*IL6r*) gene (*t* = 3.92, unadjusted *p* = 1.0 × 10^−4^). This interaction showed a trend level association in the ADNI-1 group (*t* = 1.82, unadjusted *p* = 0.06) and a very significant association in the combined dataset (*t* = 4.42, unadjusted *p* = 1.1 × 10^−5^). A second interaction was noted between this *C9* SNP and another SNP in *IL6r* (rs4240872) (*t* = 3.73, unadjusted *p* = 2.1 × 10^−4^). Similar to the first interaction, this association was found to be significant only in the combined sample (*t* = 4.15, unadjusted *p* = 3.7 × 10^−5^; ADNI-1, *t* = 1.85, unadjusted *p* = 0.06).

**Fig. 1:**
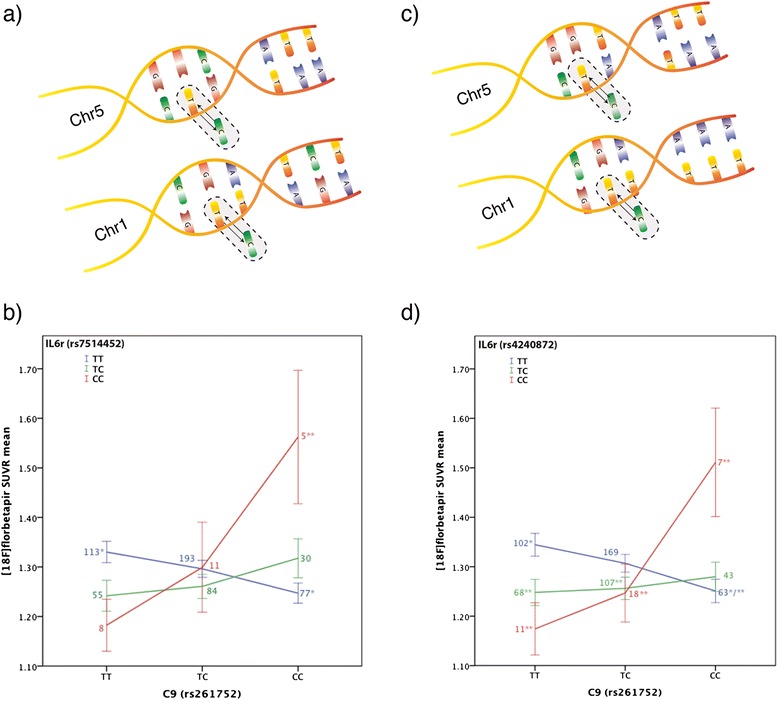
Interaction between *C9* and *IL6r* genes. **a** Representation of the SNP rs261752 (*C9* gene) in chromosome 5 and SNP rs7514452 (*IL6r* gene) in chromosome 1. **b** *C9* × *IL6r* (rs7514452) on amyloid deposition. The interaction between *C9* and *IL6r* genes is associated with amyloid burden. *Error bars* represent the standard error. *Small numbers* represent the subsample size for each genotype combination. *The mean SUVR for the assigned genotypes are different between each other (*p* = 0.05 (two-tailed)). **The mean SUVR for this genotype is higher than all other genotypes (*p* values ≤ 0.05 (two-tailed)). *p* values are adjusted according to Tukey’s HSD test. **c** Representation of the SNP rs261752 (*C9* gene) in chromosome 5 and SNP rs4240872 (*IL6r* gene) in chromosome 1. **d** *C9* × *IL6r* (rs4240872) on amyloid deposition. The interaction between *C9* and *IL6r* genes is associated with amyloid burden. *Error bars* represent the standard error. *Small numbers* represent the subsample size for each genotype combination. *The mean SUVR for the assigned genotypes are different between each other (*p* = 0.04 (two-tailed)). **The mean SUVR for the genotype CC(*C9*)*CC(*IL6r*) is higher than all other assigned genotypes (*p* values ≤0.05 (two-tailed)). p values are adjusted according to Tukey’s HSD test

Group comparisons between genotypes showed similar results for the two interactions reported. Interestingly, the *IL6r* and *C9* SNP interaction showed that, despite being not AD and having only one *ApoE ε4*-positive subject, the combination of both minor alleles (CC(*C9*)*CC(*IL6r*)) was associated with higher mean SUVR values when compared to almost all other genotype combinations (see Fig. 1. For post hoc results, see Additional file 2: Table S3).

In order to assess whether the interaction was specific to individuals in the AD spectrum, we stratified the individuals in the CN and AD spectrums (MCI and dementia phase). Both groups presented similar results than those obtained using the entire sample (see Table 3).

**Table 3:** Interactions tested within diagnostic groups in the combined dataset

	Cognitively normal (CN)				AD spectrum (MCI + AD)		
Gene interactions	SNP interactions	*β* value	*p* value	MAF	*β* value	*p* value	MAF
C9* *IL6r*	rs261752* rs7514452	0.07	0.02	0.42 0.20	0.10	1.2 × 10^−4^	0.45 0.20
C9* IL6r	rs261752* rs4240872	0.07	0.02	0.42 0.25	0.09	3.3 × 10^−4^	0.45 0.24

*MAF* minor allele frequency

### Voxel-based analysis revealed that the epistasis is related to amyloid deposition in AD-related brain regions

**Fig. 2:**
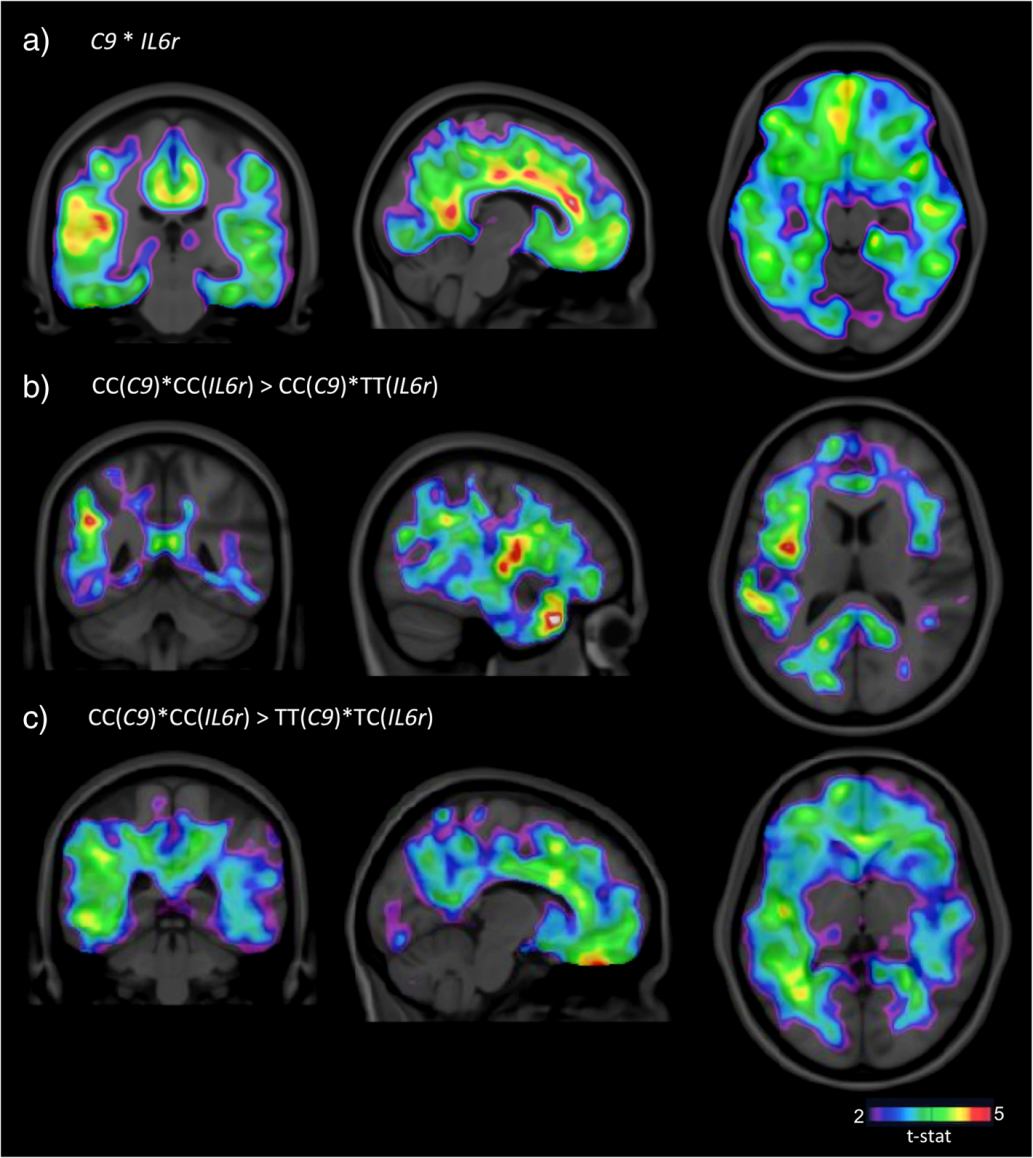
*T*-maps of contrasts between genotypes. **a** *T*-statistical parametric maps (SPM) superimposed on an average structural MRI show brain regions with high SUVR values in carriers of the genetic interaction between the SNP rs261752 (*C9* gene) and SNP rs7514452 (*IL6r* gene). Statistical differences overlap with brain regions vulnerable to AD pathophysiology. **b** SPM superimposed on an average structural MRI show the *t*-statistical contrast (CC(*C9*)*CC(*IL6r*) > CC(*C9*)*TT(*IL6r*)). Carriers of CC(*C9*)*CC(*IL6r*) have higher [^18^F]florbetapir SUVR in the frontal, parietal, and temporal cortices. **c** SPM superimposed on a structural MRI show the *t*-statistical contrast (CC(*C9*)*CC(*IL6r*) > TT(*C9*)*TC(*IL6r*)). CC(*C9*)*CC(*IL6r*) carriers have high [^18^F]florbetapir SUVR in brain regions typically affected by amyloidosis in Alzheimer’s disease. The analyses were adjusted for gender, diagnostic status, and *ApoE ε4*. The *T* value threshold of significance after RFT correction is ≥3.2 (*p* ≤ 0.05)

The voxel-based analysis showed that the interaction between *C9* and *IL6r* SNPs is associated with amyloid load in the anterior and posterior cingulate, temporal, and inferior parietal cortices bilaterally (see Fig. 2). Additionally, voxel-wise comparisons revealed that homozygous subjects for both minor alleles, when compared to either carriers of the genotype CC(*C9*)*TT(*IL6r*) or the genotype TT(*C9*)*TC(*IL6r*), have more amyloid load in the brain regions mentioned above. These differences are corrected for multiple comparisons (at 0.05 level).

### CSF biomarkers of AD neurodegeneration replicated the results obtained using the [^18^F]florbetapir SUVR

**Fig. 3:**
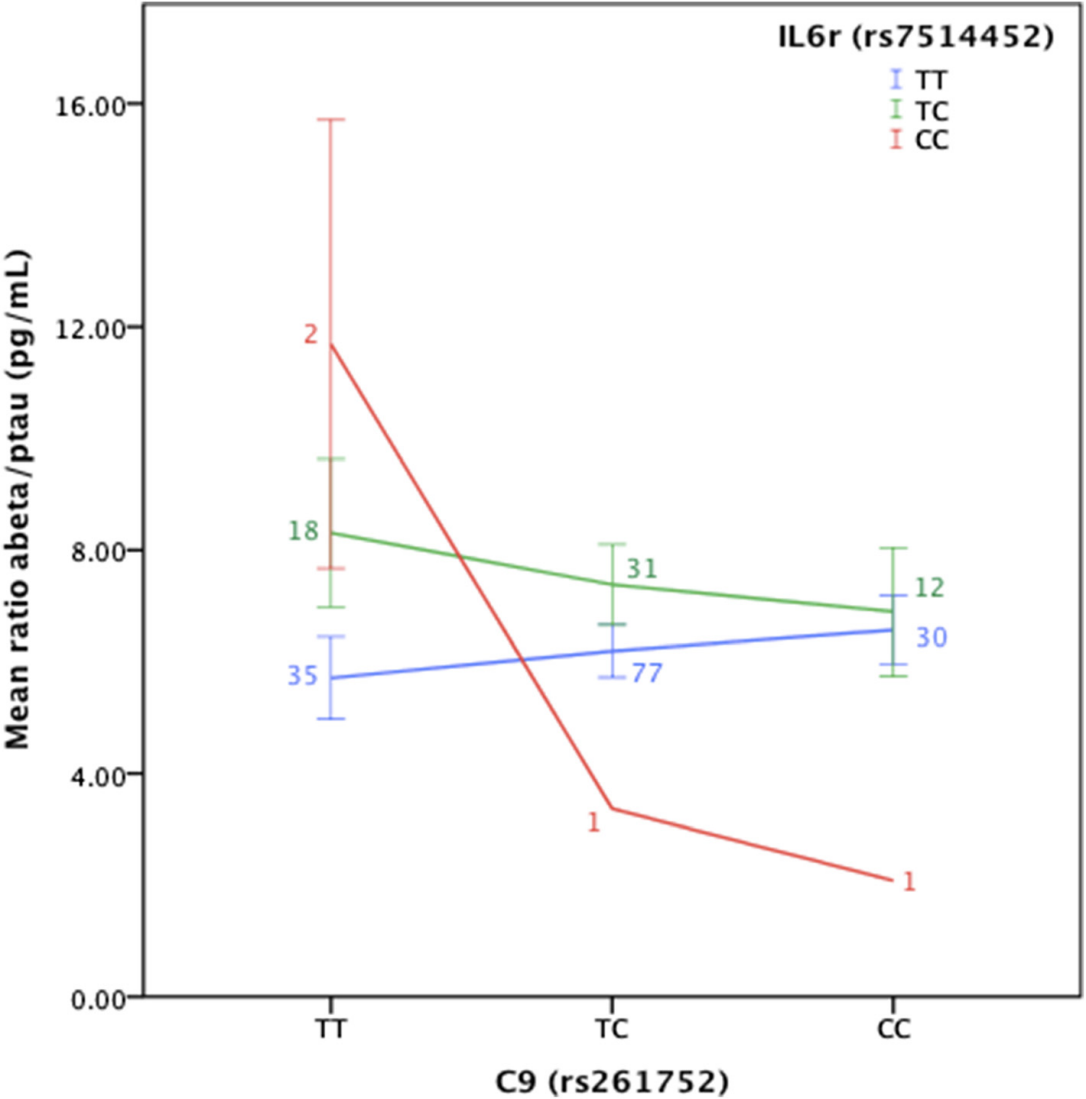
Interaction between C9 and IL6r genes using the CSF Aβ_1-42_/p-tau ratio. The interaction between *C9* and *IL6r* genes is associated with amyloid burden. *Error bars* represent the standard error. *Small numbers* represent the subsample size for each genotype combination

A subsample of 208 subjects (85 CN, 113 MCI, and 10 AD) who had baseline CSF measures was used to confirm the interaction model. The Aβ_1-42_/p-tau ratio was used as a dependent variable (CN average = 7.73, MCI average = 6.05, AD average = 2.76; difference between all groups statistically significant *p* = 5.3 × 10^−5^). The interaction analysis was replicated in the tested pair of SNPs (rs261752*rs7514452 *t* = −2.82, *p* = 0.005) (Additional file 2: Table S4). Similar observations to the findings using [^18^F]florbetapir were found; homozygous subjects for both minor alleles tend to have the lowest Aβ_1-42_/p-tau ratio (Fig. 3). However, due to sample size restrictions, group comparisons could not be performed.

### Plasma and CSF levels of IL6R protein were associated with the genetic polymorphisms

The *IL6r* genotypes of both SNPs were associated with plasmatic levels of IL6r protein (rs7514452 *t* = −2.42, unadjusted *p* = 0.01; rs4240872 *t* = −2.94, unadjusted *p* = 0.003), which shows carriers of the minor alleles having a lower level of the protein compared to non-carriers. Due to the sample size, it was not possible to verify if the same effect is present within diagnostic groups; therefore, the diagnosis was used as a covariate in the analysis. Similarly, CSF levels of IL6r protein were associated with rs4240872 (*t* = −3.17, unadjusted *p* = 0.002) but not with rs7514452 (*t* = −1.52, unadjusted *p* = 0.12). Unfortunately, there is no data available to date reflecting plasmatic levels of C9 protein that would permit us to do the correspondent analysis with the SNP rs261752.

## Discussion

In the present study, two interactions between two immune-related genes, *C9* and *IL6r*, were found to be associated with [^18^F]florbetapir SUVRs. This result suggests that Aβ burden in the brain may be differentially affected depending on the allelic combination of the cited variants.

The SNP rs261752 is an intronic variation of the *C9* gene, with no previously reported association to any phenotypic feature or neurodegenerative endophenotype. However, it has been associated with age-related macular degeneration, a highly frequent disorder among AD patients [46, 47]. Moreover, several studies have described increased immunoreactivity of classical complement molecules, including C9, in the vicinity of brain Aβ aggregates [25, 48, 49]. C9 protein is also a component of the MAC, which is responsible for disrupting cellular homeostasis, causing cell death following activation of the complement pathway [50]. Indeed, it is well known that extracellular Aβ triggers the complement cascade, leading to MAC formation [26, 48, 51]. Since MAC requires a lipid bilayer structure to act upon, it binds to the surrounding neurites [26, 52], leading to neurodegeneration and cell death. Furthermore, the protein clusterin, encoded by the AD-related CLU gene, has been shown to play an important role in reducing inappropriate MAC activity tied to physical interaction with the C9 protein [53].

The two SNPs from the *IL6r* gene are more than 1800 bp apart from each other (*r*^2^ = 0.69) and, despite not being in high linkage disequilibrium, might reflect the same signal. The SNP rs4240872 is an intronic variant of the *IL6r* gene while the variant rs7514452 is located in the 3’-untranslated region (3’-UTR), an important sequence at the end of the messenger RNA (mRNA) known to affect post-translational regulation and subsequent protein expression [54]. A previous study suggested a possible association between 3’-UTR markers and diabetes mellitus type 2 [55], an association of possible relevance owing to evidence showing that insulin signaling is down-regulated in AD (for review, see [56]). Additionally, Walston et al. [57] reported that some *IL6r* SNPs are associated with plasmatic levels of interleukin 6 (IL6), a cytokine that plays an important role in the regulation of neuroimmune responses, promoting both pro-inflammatory and anti-inflammatory effects [58–60]. Similar results were reported here showing that CSF levels of IL6r are associated with one *IL6r* SNP while plasmatic levels are associated with both SNPs (rs7514452 and rs4240872) in ADNI-1 subsample, reflecting a genotype-phenotype effect. The IL6r protein is either a part of the ligand-binding receptor of IL6 or a soluble form (s-IL6r), which binds to IL6 to enhance its activity [61, 62]. Deregulation of immune response signaling in AD is evidenced by altered protein expression in the brain [63, 64]. Differences in CSF and serum levels of both IL6 and s-IL6r are also evident when comparing AD patients to CN [65–67].

Voxel-based findings revealed by this study further corroborate global increases of amyloid load in regions typically affected by AD pathophysiology. Homozygous subjects for minor alleles of both *IL6r* and *C9* genes show higher levels of amyloid in brain areas that correspond to regions impaired in AD [68]. Interestingly, amyloid plaques depicted by amyloid imaging agents are typically surrounded by neuroinflammatory changes such as astrocytosis and microglial activation (for review, see [69]), reinforcing a link between amyloidosis and immune response. Additionally, one could claim that a reduction in the IL6r levels causes decreases in the IL6 activity, contributing to Aβ accumulation through different possible mechanisms.

In agreement with [^18^F]florbetapir findings, the interactions between *C9* and *IL6r* genes were also associated with the CSF Aβ_1-42_/p-tau ratio. This finding based on an independent measurement of brain amyloidosis provides additional evidence that *C9* and *IL6r* interactions affect brain accumulation of neuritic plaques in a disease-specific manner [70]. However, it is important to take the reduced sample size present in the CSF population into consideration.

Based on our results, it seems plausible that a combination of gene polymorphisms in complement factors and interleukins plays a synergic role in determining amyloid burden in the brain. Specifically, a particular combination of genotypes that up-regulate both C9 and IL6r may exert an additive effect via neuroinflammatory processes. Besides the supposition of how these SNPs may jointly affect amyloid accumulation in the brain, no relationship between these two genes or proteins has been reported to date with respect to amyloid metabolism. However, it has been shown that the protein IL6 is able to stimulate C9 mRNA expression in post-mortem human astrocytes and neuroblastoma cells [71, 72], showing a metabolic link between the two proteins in the cells of the nervous system.

In order to overcome the well-known limitations of association studies, several assumptions need to be addressed. For example, although all the cited proteins are related to the immune system, their roles in Aβ accumulation remain unclear. Presently, the functions of the reported SNPs remain elusive due to the lack of relevant literature. Regarding the association found between IL6r levels and *IL6r* SNPs, linking the genotype with the phenotype, it is important to mention that [73] (1) protein levels were measured on average 55 months prior to [^18^F]florbetapir image acquisition; (2) there was no association between the use of anti-inflammatory drugs and IL6r levels in this sample; and (3) beyond the effect that *IL6r* SNPs can have at the protein level, it is very important to know the effect of the *C9* genotypes on C9 protein to better understand how they jointly impact the immune response.

Among the limitations of the study, the ADNI is a cohort mostly composed of non-Hispanic Caucasians, limiting the extrapolation of the present findings to other population groups. A wider range of subjects varying in terms of ethnicity, family history, and disease progression should be considered for future replication of this study. It is also important to acknowledge that, despite postulated that amnestic MCI have high probability to convert to dementia due to AD, a considerable proportion of these individuals remain stable or convert to non-AD dementias [74], being a methodological limitation to the study of the AD spectrum. Moreover, currently, it is thought that the Aβ oligomers (soluble forms) are the most synaptotoxic (for review, see [75]) and the most chased by the immune system; however, it is not possible to detect these forms in vivo using brain imaging; [^18^F]florbetapir is only able to bind to amyloid plaques. Recently, MRI probes for targeting Aβ oligomers have been developed and will likely provide further information regarding the association between Aβ oligomers and the immune system [76]. In fact, more studies are needed to address the biological mechanisms in which gene interactions may affect the phenotype, using both amyloid plaque and Aβ oligomer quantifications.

It is also important to mention that statistical analyses between genetic factors do not define their biological interactions or interferences [21], necessitating more investigation. It should be noted that age showed negligible or no effect in our analyses and does not alter the conclusions if added in the model. Though ADNI-1 data was used to confirm significant associations, the reduced sample size could have been a limiting factor with respect to the achievement of statistical significance. Based on the effect size of the interactions found in the first analysis with ADNI-GO/2 data (data not shown), the sample size required to reach 95 % of power and a type I error of 0.05 is 497 subjects. For this reason, a less strict FDR correction was adopted in the first step of the analysis. Sample size requirements might also explain why it was not possible to fully replicate the results using data from ADNI-1, while results were replicated in the combined sample—the *p* values obtained for the interactions in the combined sample would still be significant at 0.05 level if FDR correction had to be applied. Additionally, the highly significant *p* values obtained with the combined sample indicate a high likelihood that the initial results obtained using ADNI 2 data were not a consequence of a type II error.

## Conclusions

In conclusion, using a clinically well-characterized and genetically homogenous sample, as well as a confirmatory imaging analysis, our hypothesis-driven analyses identified several epistatic links between *IL6r* and *C9* genes, suggesting genetic components linking the immune system and brain amyloidosis. Though further studies are required, these results suggest that these interacting genotypes may represent potential biomarkers for differential treatment of AD.

## Additional files

Additional file 1: Figure S1. [^18^F]Florbetapir SUVR analytical method. Flowchart showing acquisition methods (purple), image processing (blue), and outcomes (green). *PET* positron emission tomography, *MRI* magnetic resonance imaging, *GM* gray matter, *WM* white matter, *CSF* cerebrospinal fluid, *FWHM* first width half maximum, *ROI* region of interest. (TIFF 2485 kb) Additional file 2: Tables S1–S4. Table S1. List of SNPs within each set. Table S2. R epistasis results (first 15 lines). Table S3. Tukey’s HSD test results. Table S4. Interaction tested using Aβ_1-42_/p-tau ratio as phenotype. (DOCX 66 kb)

## Abbreviations

AD: Alzheimer’s disease
ADNI: Alzheimer’s Disease Neuroimaging Initiative
APOE: apolipoprotein E
Aβ: amyloid β
C: complement factor
CN: cognitively normal
CSF: cerebrospinal fluid
FDR: false discovery rate
GWAS: genome-wide association studies
HSD: honest significant difference
IL: interleukin
MAC: membrane attack complex
MCI: mild cognitive impairment
MINC: Medical Imaging NetCDF
MRI: magnetic resonance imaging
NFT: neurofibrillary tangle
PET: positron emission tomography
p-tau: phosphorylated tau
RFT: random field theory
SNP: single nucleotide polymorphism
SUVR: standardized uptake value ratio
