# Supplementary figures and images for: Epistasis analysis links immune cascades and cerebral amyloidosis
Source: J Neuroinflammation. 2015 Dec 1;12:227. doi: 10.1186/s12974-015-0436-z (PMC4666175; doi:10.1186/s12974-015-0436-z)

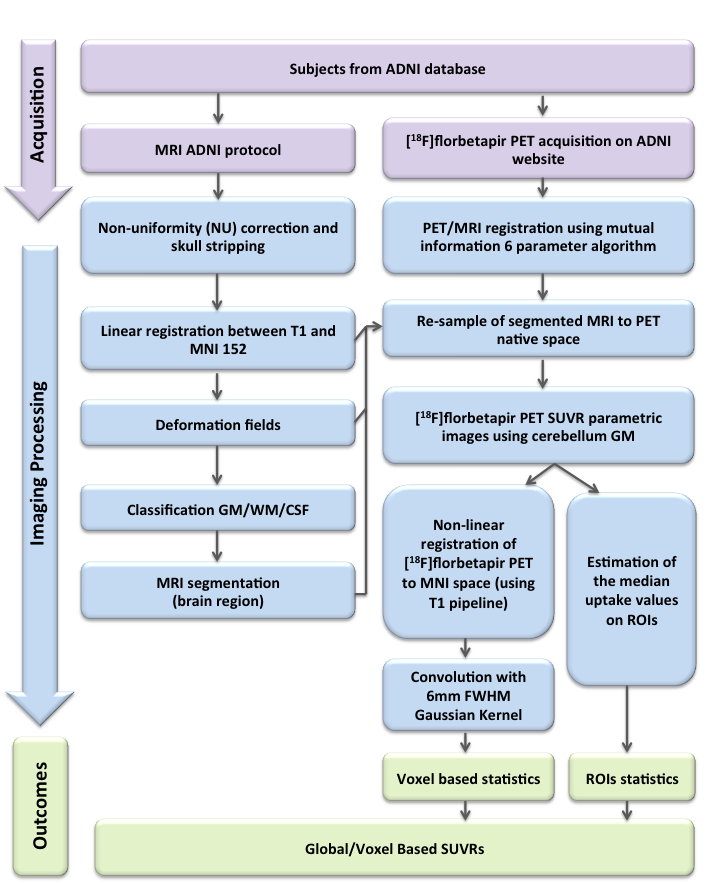

Supplement: Additional file 1: Figure S1. — [18F]Florbetapir SUVR analytical method. Flowchart showing acquisition methods (purple), image processing (blue), and outcomes (green). PET positron emission tomography, MRI magnetic resonance imaging, GM gray matter, WM white matter, CSF cerebrospinal fluid, FWHM first width half maximum, ROI region of interest. (TIFF 2485 kb) [file 12974_2015_436_MOESM1_ESM.tiff]
