# Supplementary material for: Epistasis analysis links immune cascades and cerebral amyloidosis
Source: J Neuroinflammation. 2015 Dec 1;12:227. doi: 10.1186/s12974-015-0436-z (PMC4666175; doi:10.1186/s12974-015-0436-z)
Supplement: Additional file 2: Tables S1–S4. — Table S1. List of SNPs within each set. Table S2. R epistasis results (first 15 lines). Table S3. Tukey’s HSD test results. Table S4. Interaction tested using Aβ1-42/p-tau ratio as phenotype. (DOCX 66 kb) [file 12974_2015_436_MOESM2_ESM.docx]

**Supplementary Table 1**. List of SNPs within each set.

| **Set** | **Gene** | **SNP** |
| --- | --- | --- |
| Set 1 | *IL-1β* | rs1071676, rs1143634, rs1143633, rs3136558 |
|  | *IL4* | rs2243268, rs2227282 |
|  | *IL6* | rs2069832, rs2069835, rs2069837, rs2066992 |
|  | *IL6r* | rs4845617, rs1386821, rs4845618, rs8192282, rs4537545, rs11265618, rs4240872, rs7514452 |
|  | *IL10* | rs3024496 |
|  | *IL12* | rs2243123, rs568408, rs3212227, rs11574790, rs2853694, rs2569254, rs1003199, rs2546893, rs730691 |
|  | *IL18* | rs5744256, rs2043055, rs360717 |
| Set 2 | *C5* | rs41258306, rs7045519, rs17612, rs7026551, rs2269066, rs4837805, rs10760135, rs17611, rs7027797, rs10985126, rs17220750, rs7031128, rs1468673 |
|  | *C9* | rs263275, rs11959122, rs261752, rs261753, rs696758, rs696764, rs4957473, rs835218 |

**Supplementary Table 2**. R epistasis results (firsts 15 lines).

| **SNP1^a^** | **SNP2^b^** | ***P* value^c^** | ***P* value adj^d^** |
| --- | --- | --- | --- |
|  |  |  |  |
| rs7514452 | rs261752 | 0.0001027 | 0.0669045^*^ |
| rs4240872 | rs261752 | 0.0002157 | 0.0702403^*^ |
| rs2069837 | rs17220750 | 0.0009282 | 0.2014374 |
| rs2243268 | rs7026551 | 0.0019076 | 0.3104708 |
| rs2243268 | rs7027797 | 0.0049702 | 0.5752360 |
| rs4845618 | rs41258306 | 0.0079221 | 0.5752360 |
| rs4845618 | rs17612 | 0.0080400 | 0.5752360 |
| rs5744256 | rs835218 | 0.0081852 | 0.5752360 |
| rs8192282 | rs263275 | 0.0083114 | 0.5752360 |
| rs11574790 | rs261752 | 0.0108487 | 0.5752360 |
| rs7514452 | rs696758 | 0.0114193 | 0.5752360 |
| rs11265618 | rs263275 | 0.0137167 | 0.5752360 |
| rs3136558 | rs2269066 | 0.0138882 | 0.5752360 |
| rs2243268 | rs10985126 | 0.0147935 | 0.5752360 |
| rs2227282 | rs263275 | 0.0160563 | 0.5752360 |
|  |  |  |  |

^a^ SNP1 First SNP from set 1

^b^ SNP2 Second SNP from set 2

^c^ *P* value  *P* value from R epistasis analysis

^d^ *P* value adj. FDR corrected *P* value

^*^ Interactions that reached significance after FDR correction at 0.1 level.

**Supplementary Table 3.** Tukey’s HSD test results.

| Contrasted genotypes | |  | Contrasted genotypes | |  |
| --- | --- | --- | --- | --- | --- |
| ***C9*IL6r* (rs7514452)** | ***C9* IL6r* (rs7514452)** | ***P* value** | ***C9*IL6r* (rs4240872)** | ***C9*IL6r* (rs4240872)** | ***P* value** |
| TT*TC | TT*TT | 0.17 | TT*TC | TT*TT | 0.13 |
| TT*CC | TT*TT | 0.85 | TT*CC | TT*TT | 0.55 |
| TC*TT | TT*TT | 0.69 | TC*TT | TT*TT | 0.79 |
| TC*TC | TT*TT | 0.55 | TC*TC | TT*TT | 0.16 |
| TC*CC | TT*TT | 0.96 | TC*CC | TT*TT | 0.61 |
| CC*TT | TT*TT | **0.05** | CC*TT | TT*TT | **0.04** |
| CC*TC | TT*TT | 0.99 | CC*TC | TT*TT | 0.92 |
| CC*CC | TT*TT | **0.03** | CC*CC | TT*TT | 0.24 |
| TT*CC | TT*TC | 1.00 | TT*CC | TT*TC | 0.99 |
| TC*TT | TT*TC | 0.86 | TC*TT | TT*TC | 0.79 |
| TC*TC | TT*TC | 0.99 | TC*TC | TT*TC | 1.00 |
| TC*CC | TT*TC | 1.00 | TC*CC | TT*TC | 1.00 |
| CC*TT | TT*TC | 1.00 | CC*TT | TT*TC | 1.00 |
| CC*TC | TT*TC | 0.83 | CC*TC | TT*TC | 0.98 |
| CC*CC | TT*TC | **0.001** | CC*CC | TT*TC | **0.01** |
| TC*CC | TT*CC | 0.99 | TC*CC | TT*CC | 0.90 |
| TC*TC | TT*CC | 0.99 | TC*TC | TT*CC | 0.99 |
| TC*CC | TT*CC | 0.99 | TC*CC | TT*CC | 1.00 |
| CC*TT | TT*CC | 1.00 | CC*TT | TT*CC | 1.00 |
| CC*TC | TT*CC | 0.96 | CC*TC | TT*CC | 0.96 |
| CC*CC | TT*CC | **0.01** | CC*CC | TT*CC | **0.03** |
| TC*TC | TC*TT | 0.99 | TC*TC | TC*TT | 0.90 |
| TC*CC | TC*TT | 0.99 | TC*CC | TC*TT | 0.96 |
| CC*TT | TC*TT | 0.65 | CC*TT | TC*TT | 0.48 |
| CC*TC | TC*TT | 0.99 | CC*TC | TC*TT | 1.00 |
| CC*CC | TC*TT | **0.006** | CC*CC | TC*TT | 0.06 |
| TC*CC | TC*TC | 1.00 | TC*CC | TC*TC | 1.00 |
| CC*TT | TC*TC | 0.97 | CC*TT | TC*TC | 0.99 |
| CC*TC | TC*TC | 0.99 | CC*TC | TC*TC | 0.99 |
| CC*CC | TC*TC | **0.004** | CC*CC | TC*TC | **0.02** |
| CC*TT | TC*CC | 1.00 | CC*TT | TC*CC | 1.00 |
| CC*TC | TC*CC | 0.99 | CC*TC | TC*CC | 0.99 |
| CC*CC | TC*CC | **0.01** | CC*CC | TC*CC | **0.03** |
| CC*TC | CC*TT | 0.72 | CC*TC | CC*TT | 0.90 |
| CC*CC | CC*TT | **0.001** | CC*CC | CC*TT | **0.009** |
| CC*CC | CC*TC | **0.03** | CC*CC | CC*TC | 0.07 |

*P* values ≤0.05 are highlighted in bold.

**Supplementary Table 4.** Interaction tested using Aβ_1-42_/p-tau ratio as phenotype.

| Gene Interactions | SNP Interactions | MAF^a^ | β value | *P* value |
| --- | --- | --- | --- | --- |
|  |  |  |  |  |
| *C9***IL6r* | rs261752 * rs7514452 | 0.46/0.18 | -1.98 | 5.2 x 10^-3^ |
|  |  |  |  |  |

^a^ MAF – minor allele frequency.
